# Evaluative threat as an internal dimension of music performance anxiety among Chinese university music students

**DOI:** 10.3389/fpsyg.2026.1902711

**Published:** 2026-08-19

**Authors:** Yang Yu, Yue Zhang, Wei Liu, Weiqi Liu

**Affiliations:** 1 Music Academy, Hunan Normal University, Changsha, Hunan, China; 2 School of Music and Dance, Daqing Normal University, Daqing, Heilongjiang, China; 3 Graduate School, José Rizal University, Mandaluyong, Philippines; 4 School of Arts and Media, Hunan University of Information Technology, Changsha, Hunan, China

**Keywords:** Chinese university music students, confirmatory factor analysis, evaluative threat, face-loss concerns, music performance anxiety, public performance

## Abstract

**Background:**

Music performance anxiety (MPA) is common among university music students. Although MPA has been viewed as multidimensional, the position of evaluative threat (ET) within its internal structure remains unclear in Chinese university music students. This study examined whether ET forms an internal dimension of MPA, together with anticipatory tension and error monitoring (ATEM) and anxiety-related performance impairment (ARPI).

**Methods:**

A cross-sectional online survey was conducted with 664 Chinese university music students. The 12-item measure assessed ATEM, ET, and ARPI. Data were analyzed using exploratory factor analysis, confirmatory factor analysis, reliability and validity tests, measurement invariance analysis, correlation analysis, multiple regression, and group difference analysis.

**Results:**

EFA and CFA supported a three-factor structure consisting of ATEM, ET, and ARPI. The three-factor model showed good fit and outperformed one-factor and two-factor models. All three dimensions showed acceptable reliability and validity. ET was positively correlated with ATEM and ARPI. Regression analysis showed that ATEM and ET were both positively associated with ARPI. Female students, lower-year undergraduate students, instrumental music students, and students with low public performance frequency reported higher scores.

**Conclusion:**

ET is an important internal dimension of MPA among Chinese university music students. Face-loss concerns and evaluative pressure should be considered in music performance training and psychological support.

## 1 Introduction

Stable and expressive stage performance is an important goal of performance training in university music programs. For music students, stage performance is also closely related to course assessment, performance opportunities, and professional development. Music performance anxiety (MPA) has therefore become a common educational and psychological issue in university music training (Fernholz et al., 2019; Farley and Kelley, 2023). MPA is usually manifested in physiological, cognitive, emotional, and behavioral symptoms and is especially salient in situations involving perceived threat and high self-investment (Herman and Clark, 2023). These responses may weaken students’ attentional control, physical stability, and emotional regulation, thereby affecting technical execution, stage behavior, and overall performance quality (Zakaria et al., 2013; Kaleńska-Rodzaj, 2020). High or persistent anxiety may also affect students’ academic performance, mental health, and career development (Moura and Serra, 2021; Schoonover, 2026; Hernández-Dionis et al., 2025). Therefore, MPA is an important psychological factor affecting the stage performance, learning development, and professional growth of university music students.

In the context of university music training in China, MPA needs to be understood in relation to the preparation and performance process, evaluative relationships, and perceived performance outcomes. Existing research links MPA to concern over mistakes and self-monitoring (Studer et al., 2014; Huang and Song, 2021), fear of negative evaluation and face-related concerns (Papageorgi et al., 2007; Leary, 1983; Hu, 1944; Hwang, 2011; Zane and Yeh, 2002), and lower perceived performance quality (Tanskanen, 2025). These findings point to three related components—tension and error monitoring, evaluative threat, and anxiety-related performance impairment—but whether they constitute distinct dimensions of MPA among Chinese university music students remains unclear.

In recent years, studies on MPA among Chinese music students have mainly focused on influencing factors, psychological mechanisms, and intervention pathways. Related studies have shown that social support, psychological capital, self-efficacy, flow experience, and career expectations are associated with MPA (Huawei and Jenatabadi, 2024; Jiang and Tong, 2024; Wang and Yang, 2024). A study on virtual reality (VR) exposure training among Chinese and Japanese music students also suggests that cultural background and educational environment may influence the manifestation and intervention effects of MPA (Zhang et al., 2026). However, direct examination of the internal structure of MPA among Chinese university music students and the relationships among its dimensions remains insufficient.

Based on the above analysis, this study focuses on Chinese university music students and examines the internal structure of MPA and the relationships among its dimensions. This study conceptualizes MPA as consisting of three dimensions: anticipatory tension and error monitoring (ATEM), evaluative threat (ET), and anxiety-related performance impairment (ARPI). ATEM reflects students’ tension, concern over mistakes, and self-monitoring before and during performance. ET reflects students’ concerns about public evaluation, ability image, professional recognition, and loss of face. ARPI reflects students’ perceived interference of anxiety with technical control, performance quality, and musical expression. This study examines the relationships among ATEM, ET, and ARPI and compares differences across gender, year level, major direction, and public performance frequency. The study aims to clarify whether ATEM, ET, and ARPI can constitute important internal dimensions of MPA among Chinese university music students and to provide evidence for psychological support and educational intervention in music performance training.

## 2 Literature review and hypotheses

### 2.1 MPA as a three-dimensional structure

Previous studies have provided a basis for understanding MPA as a multidimensional construct. Kenny (2009) examined the factor structure of the Kenny Music Performance Anxiety Inventory (K-MPAI) and showed that MPA involves somatic anxiety, negative cognition, self- and other-scrutiny, concerns about performance outcomes, memory reliability, and psychological vulnerability. Papageorgi et al. (2007) also explained the development of MPA from the perspectives of individual characteristics, task characteristics, performance environment, and cognitive appraisal. These studies indicate that MPA is a multidimensional psychological structure involving physical responses, cognitive appraisal, evaluative pressure, and performance outcomes. Student musicians provide an important context for examining this structure, because performance anxiety can appear at an early stage of music training and affect students’ learning experiences and stage performance (Osborne and Kenny, 2005). Research on undergraduate music students has also shown that MPA is associated with physiological responses, cognitive concerns, emotional experiences, behavioral responses, and educational environments (Barros et al., 2022). Recent validation studies among Chinese music students further suggest that the structure of MPA needs to be examined in relation to specific cultural and training contexts (Zhao and Wong, 2026; Pan et al., 2025).

In university music training, these multidimensional features can be organized around three related domains. Evidence concerning pre-performance tension, concern over mistakes, and self-monitoring supports ATEM (Studer et al., 2014; Huang and Song, 2021; Burin and Osório, 2017). Research on performance environment, fear of negative evaluation, and face-related concerns supports ET (Papageorgi et al., 2007; Leary, 1983; Hu, 1944; Hwang, 2011; Zane and Yeh, 2002). Findings linking MPA to lower perceived performance quality support ARPI (Nielsen et al., 2018; Tanskanen, 2025). Together, these lines of evidence provide a basis for testing whether ATEM, ET, and ARPI form a coherent three-dimensional structure.

Accordingly, this study organizes these components into three testable dimensions-ATEM, ET, and ARPI-within the context of university music training. This organization brings together previously dispersed evidence concerning tension responses, evaluative concerns, and perceived performance impairment. Therefore, this study proposes the following hypothesis:

*H1*: MPA can be identified as a three-dimensional structure consisting of ATEM, ET, and ARPI.

### 2.2 ET in music performance contexts

Music performance has a clear public and evaluative nature. Theories of social anxiety and self-presentation suggest that anxiety tends to arise when individuals wish to create a favorable impression but doubt whether they can do so successfully, while fear of negative evaluation reflects sensitivity to others’ negative judgments (Schlenker and Leary, 1982; Leary, 1983; Carleton et al., 2011). Studies of examination situations, audience presence, and subjective performance quality have shown that public evaluation can influence both anxiety experiences and perceived performance quality (Iusca and Dafinoiu, 2012; Nielsen et al., 2018; Guyon et al., 2022). Social expectations and external evaluative pressure may also be transformed into MPA through cognitive processes such as rumination (Yan and Liu, 2026). Accordingly, when students interpret evaluations from teachers, peers, judges, or audiences as judgments of their ability and professional image, evaluative pressure may become an internal ET component of MPA.

In university music training in China, ET also has a culturally specific expression. The concepts of “lian” and “mianzi” concern social reputation, recognition from others, and relational standing (Hu, 1944; Hwang, 2011), and loss of face can function as a culturally grounded variable in psychological assessment (Zane and Yeh, 2002). Research in educational contexts further links performance to family expectations, social networks, and recognition from others (Guan and Ploner, 2020; Prutskikh and Merkulova, 2022). Thus, public mistakes may be experienced not only as technical failures but also as threats to professional image and face. In this study, concern over loss of face is therefore treated as a cultural expression of ET rather than as a separate construct.

Based on this analysis, stronger ET may be associated with greater mistake monitoring and perceived anxiety-related performance impairment. Therefore, this study proposes the following hypothesis:

*H2*: ET is significantly and positively correlated with both ATEM and ARPI.

### 2.3 ATEM, ET, and ARPI

Attentional control theory provides an important explanation for understanding how anxiety affects performance. According to this theory, anxiety weakens goal-directed attentional control and increases attention to threat-related information (Eysenck et al., 2007). From the perspective of executive function, this effect is mainly reflected in inhibition and shifting. Inhibition helps individuals exclude information that is irrelevant to the current task, whereas shifting helps individuals flexibly switch between different task goals and attentional objects (Miyake et al., 2000). Music performance requires students to continuously mobilize working memory, motor control, and emotional regulation (Zhang, 2026). During instrumental or vocal performance, students need to maintain pitch, rhythm, memory, bodily movement, and musical expression at the same time (Chenette, 2021; Toader et al., 2023). Therefore, stable attentional control is an important condition for successful performance.

The association between ATEM and ARPI can be understood from the perspective of attentional control. Pre-performance tension, concern over mistakes, and self-monitoring may increase students’ attention to internal anxiety cues. Previous studies have shown that the cognitive components of MPA often include worry, negative thoughts, attentional distraction, and excessive focus on mistakes, which can interfere with task-related thinking and behavioral performance during performance (Sinden, 1999; Castro, 2003; Kuan, 2012). When students continuously focus on possible mistakes, they may find it more difficult to inhibit distracting information that is irrelevant to the current musical task. At this point, working memory resources may shift from the musical task itself to error monitoring (Maidhof, 2013). Students may also find it more difficult to switch flexibly between technical control, musical expression, and on-stage adjustment. Therefore, the association between ATEM and students’ perceived interference with technical control and musical expression may reflect the diversion of attentional resources toward error monitoring.

The association between ET and ARPI can also be understood from the perspective of attentional bias. In music performance, evaluative pressure may lead students to pay more attention to others’ reactions and their own performance state. When students interpret performance mistakes as signs of insufficient ability, damaged professional image, or reduced social recognition, they may be more likely to shift attentional resources toward external evaluation. This attentional bias may weaken their engagement with the musical task itself and increase their perception of decreased performance quality. Recent research has also shown that MPA is closely associated with perfectionism and fear of negative evaluation (Gök and Yalçınkaya-Alkar, 2025).

ARPI does not emphasize general concerns about performance outcomes. Rather, it emphasizes students’ subjective judgment that anxiety interferes with performance. Nielsen et al. (2018) found that MPA was associated with lower subjective performance quality and post-event rumination. This suggests that MPA is associated not only with the performance process but also with students’ evaluations of their own performance quality. Thus, error monitoring and evaluative threat provide a theoretical basis for expecting positive associations of ATEM and ET with perceived performance impairment. Based on the above theoretical and empirical evidence, this study proposes the following hypothesis:

*H3*: When ATEM and ET are included simultaneously in the regression model, both will show significant positive associations with ARPI.

### 2.4 Group differences

MPA may vary according to student characteristics and training experience. Gender, year level, major direction, and public performance frequency reflect individual characteristics, training stage, task type, and performance exposure, respectively. Previous studies have shown that gender, instrument type, performance context, and training stage are associated with MPA levels (Iusca and Dafinoiu, 2012; Barros et al., 2022; Papageorgi, 2022). Studies on Chinese music students also suggest that social support, psychological capital, self-efficacy, and career expectations are related to MPA manifestations (Huawei and Jenatabadi, 2024; Jiang and Tong, 2024; Wang and Yang, 2024).

These studies indicate that MPA is related to students’ individual resources, training experience, and developmental context. However, existing studies differ in sample characteristics, professional backgrounds, and measurement tools, and there is still insufficient comparison of group differences across specific dimensions. This study therefore does not propose a single directional hypothesis. Instead, it treats group differences as a research question. This approach allows a more cautious examination of how ATEM, ET, and ARPI vary across students with different genders, year levels, major directions, and public performance frequencies.

Comparing group differences in ATEM, ET, and ARPI can help identify student groups that may need more performance-related psychological support. Based on this, this study proposes the following research question:

*RQ1*: Do ATEM, ET, and ARPI differ by gender, year level, major direction, and public performance frequency?

**Figure 1:**
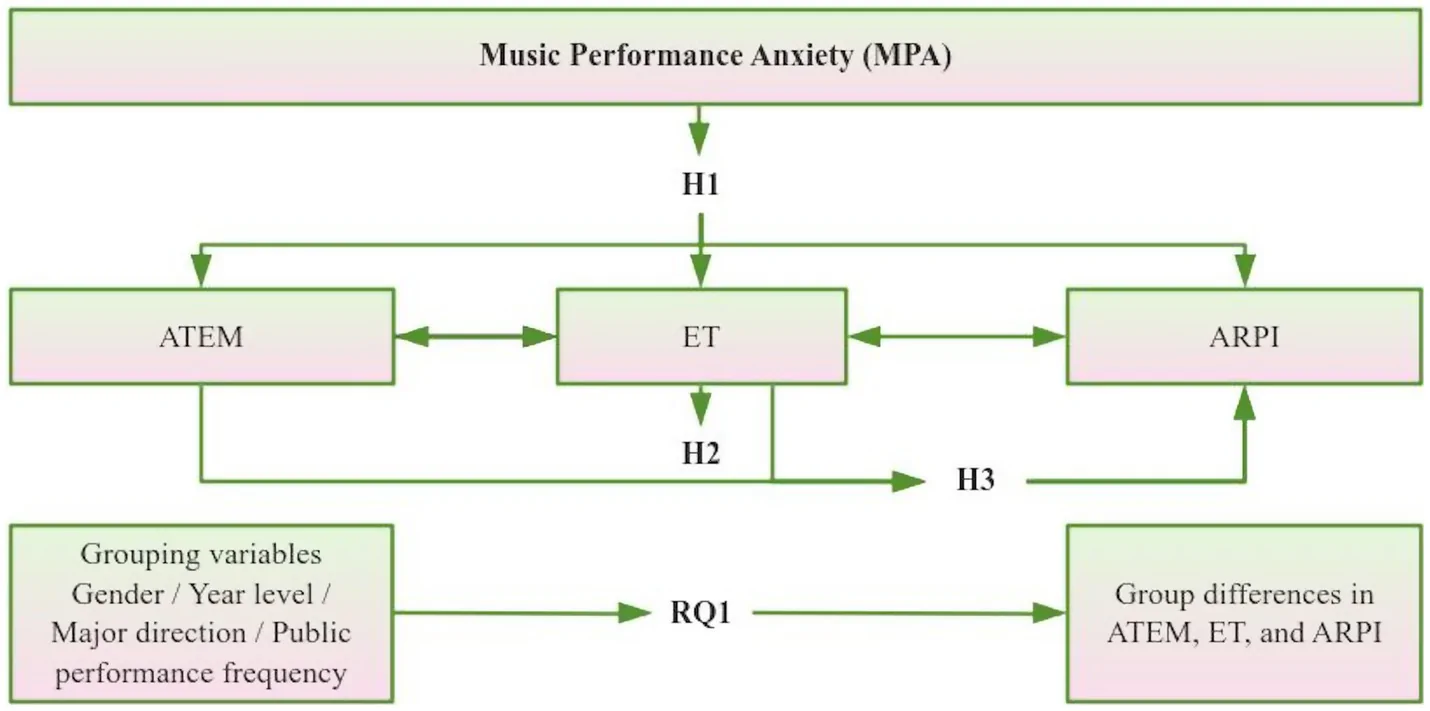
Conceptual framework of the study. The framework shows the three-dimensional structure of MPA, the hypothesized correlations of ET with ATEM and ARPI, the hypothesized associations of ATEM and ET with ARPI, and group differences across demographic and performance-related variables.

Figure 1 presents the conceptual framework of this study, including the three-dimensional structure of MPA, the hypothesized associations of ATEM and ET with ARPI, and the analysis of group differences in ATEM, ET, and ARPI.

## 3 Materials and methods

### 3.1 Participants and survey procedure

This study used a cross-sectional questionnaire design. The participants were recruited primarily from students enrolled in music and music-related programs at Hunan University of Information Technology in Changsha, Hunan Province, China. Approximately 900 survey invitations were distributed, and data were collected from March 6 to May 29, 2026. After data screening, 664 valid questionnaires were retained and included in the subsequent statistical analyses. The sample covered different year levels and major directions, including vocal music, instrumental music, composition/conducting, and music education or other related areas. The questionnaire also collected information on gender, year level, major direction, and public performance frequency for subsequent group difference analyses.

Data were collected through an anonymous online self-report questionnaire administered through Wenjuanxing. A QR code linked to the questionnaire was displayed on the survey cover page, and students participated voluntarily by scanning the QR code with their mobile phones. Before completing the questionnaire, participants read the study information and informed consent statement. The statement explained the purpose of the study, voluntary participation, anonymity, data use, and the right to withdraw. Participants could proceed with the questionnaire only after providing informed consent. The questionnaire did not collect names, student ID numbers, contact information, identification numbers, IP addresses, or any other personally identifiable information.

During data screening, questionnaires with incomplete responses, duplicate submissions, identical responses to all items, or clearly insufficient response time were excluded. All participants were adults aged 18 years or older. This study was an anonymous, voluntary, non-interventional educational questionnaire survey. The research process followed the basic ethical principles for studies involving human participants and met the requirements for low-risk anonymous questionnaire research.

### 3.2 Measurement instrument

The questionnaire consisted of two parts. The first part collected participants’ basic information, including gender, year level, major direction, and public performance frequency. The second part measured experiences related to MPA. The MPA items were context-specific items developed on the basis of previous MPA research, research on fear of negative evaluation, and the public performance context of Chinese university music students. The item content covered pre-performance tension, physiological arousal, concern over mistakes, self-monitoring, others’ evaluations, ability image, embarrassment or loss of face after public mistakes, and the influence of anxiety on performance quality. After the initial items were developed, three experts with experience in music education, music performance teaching, and psychometric research were invited to review them. The experts mainly examined whether the items covered the research construct and whether they were suitable for the performance training context of Chinese university music students. Based on expert feedback, the wording of some items was revised.

The final MPA measure consisted of 12 items. The 12 MPA items were rated on five-point response scales, with responses scored from 1 to 5 and higher scores indicating stronger MPA-related experiences. According to the theoretical framework, the 12 items corresponded to three expected dimensions. ATEM included five items, namely Items 5–9, reflecting tension, physiological arousal, fear of making mistakes, and self-monitoring before and during performance. ARPI included three items, namely Items 10–12, reflecting the interference of anxiety with performance quality, technical control, and musical expression. ET included four items, namely Items 13–16, reflecting students’ concerns about others’ evaluations, ability image, and embarrassment or loss of face after public mistakes.

### 3.3 Data analysis

Data analysis was conducted using SPSSAU^1^, Python, and JASP 0.98.1. The analysis mainly included the following steps. First, valid questionnaires were screened, and the means, standard deviations, skewness, and kurtosis of each item and main study variable were calculated. Second, Harman’s single-factor test was used as an initial diagnostic test for common method bias, and the fit of the single-factor model was also considered as supplementary evidence. This method is commonly used to examine common method bias in cross-sectional self-report questionnaire studies (Podsakoff et al., 2003). The Harman test and single-factor model results were treated only as diagnostic evidence.

Third, to examine the internal structure of MPA, the 664 participants were randomly divided into two equal subsamples. One subsample was used for exploratory factor analysis (EFA), and the other was used for confirmatory factor analysis (CFA), with each subsample including 332 participants. Before EFA, the Kaiser–Meyer–Olkin (KMO) value and Bartlett’s test of sphericity were used to determine whether the data were suitable for factor analysis. Because the dimensions of MPA were theoretically expected to be correlated, this study fixed the extraction at three factors and used Promax oblique rotation.

Fourth, CFA was used to further examine the measurement model. Model comparisons included a one-factor model, a two-factor model, and a three-factor model. Model fit was evaluated using *χ*^2^/*df*, the comparative fit index (CFI), the Tucker-Lewis index (TLI), the root mean square error of approximation (RMSEA), and the standardized root mean square residual (SRMR). In general, *χ*^2^/*df* below 3, CFI and TLI close to or above 0.90, RMSEA below 0.08, and SRMR below 0.08 indicate acceptable model fit (Hu and Bentler, 1999).

Fifth, reliability and validity were examined. Internal consistency was evaluated using Cronbach’s *α*. Composite reliability (CR) was used to assess the consistency of latent variables, and average variance extracted (AVE) was used to evaluate convergent validity. Discriminant validity was examined using the Fornell-Larcker criterion by comparing the square root of AVE for each construct with the correlations among constructs (Fornell and Larcker, 1981). Cronbach’s α and CR above 0.70 and AVE above 0.50 were considered acceptable.

Sixth, Pearson correlation analysis was used to examine the correlations among ATEM, ET, and ARPI to address H2. Seventh, multiple linear regression analysis was conducted to examine the relationships of ATEM and ET with ARPI to test H3. Before regression analysis, the variance inflation factor (VIF) was used to test multicollinearity, with VIF values below 5 considered acceptable.

Eighth, multigroup confirmatory factor analysis was conducted in JASP to examine measurement invariance across gender, undergraduate year level, major direction, and public performance frequency. Configural, metric, and scalar invariance were tested sequentially. Invariance was considered supported when the decrease in CFI did not exceed 0.010, the increase in RMSEA did not exceed 0.015, and the increase in SRMR did not exceed 0.030 for metric invariance or 0.010 for scalar invariance (Chen, 2007). Gender invariance was tested between male and female students, year-level invariance across first- to fourth-year undergraduates, major-direction invariance between vocal and instrumental music students, and performance-frequency invariance across low-, medium-, and high-frequency groups. The smaller composition/conducting and music education or other related groups were excluded from the major-direction analysis because of unstable model estimates.

**Figure 2:**
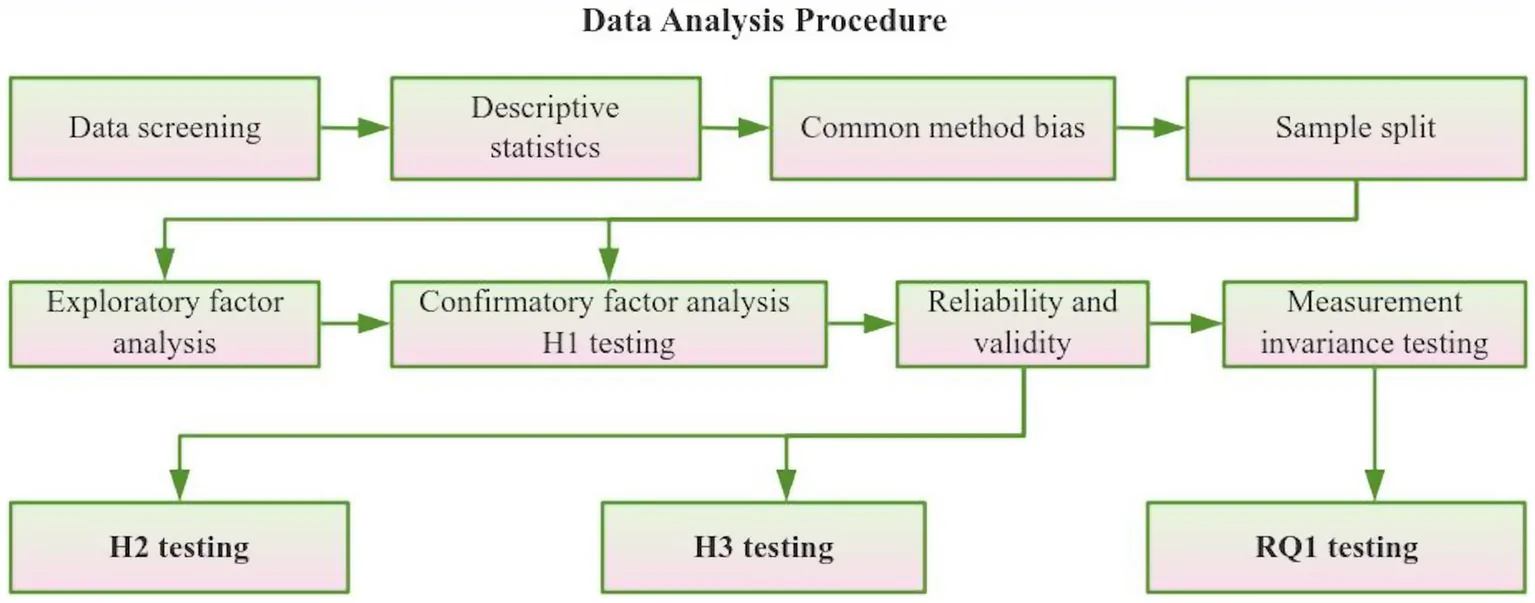
Presents the data analysis procedure, including data screening, EFA, CFA, reliability and validity testing, measurement invariance testing, correlation analysis, regression analysis, and group difference analysis for testing H1–H3 and addressing RQ1.

Finally, to address RQ1, gender differences and major-direction differences between vocal music and instrumental music students were examined using independent-samples t-tests. When the assumption of homogeneity of variance was not met, Welch’s t-test was used. Differences by year level and public performance frequency were examined using one-way analysis of variance. When the assumption of homogeneity of variance was met, ordinary one-way ANOVA was used. When the assumption was not met, Welch’s ANOVA was used. Cohen’s d was reported as the effect size for t-tests, and eta squared (*η*^2^) was reported as the effect size for ANOVA. Significant omnibus ANOVA results were followed by Games–Howell *post hoc* comparisons when the homogeneity-of-variance assumption was not met and Tukey’s HSD comparisons when the assumption was met. Multiple comparisons were controlled using the adjustments incorporated in the Games–Howell and Tukey HSD procedures. The data analysis procedure is shown in Figure 2.

## 4 Results

### 4.1 Sample characteristics and common method bias test

A total of 664 valid questionnaires were included in this study. The basic sample information is presented in Table 1. There were 217 male participants, accounting for 32.68%, and 447 female participants, accounting for 67.32%. The sample covered students from the first year to the postgraduate level, with first-year, second-year, and third-year students representing a relatively large proportion. In terms of major direction, vocal music and instrumental music were the main groups, accounting for 56.48 and 33.73%, respectively. In terms of public performance frequency, students with low public performance frequency accounted for the largest proportion, at 66.72%.

**Table 1:** Basic sample information.

Variable	Category	Frequency	Percentage
Gender	Male	217	32.68%
	Female	447	67.32%
Year level	First year	201	30.27%
	Second year	167	25.15%
	Third year	220	33.13%
	Fourth year	75	11.30%
	Postgraduate	1	0.15%
Major direction	Vocal music	375	56.48%
	Instrumental music	224	33.73%
	Composition/conducting	28	4.22%
	Music education or other related areas	37	5.57%
Public performance frequency	Low	443	66.72%
	Medium	187	28.16%
	High	34	5.12%

Because this study used cross-sectional self-report questionnaire data, common method bias was examined. Harman’s single-factor test showed that the first unrotated factor explained 62.785% of the total variance, which was higher than the commonly used 50% threshold. This suggested the possibility of common method bias or high item homogeneity. Further CFA model comparison showed that the one-factor model had poor fit, *χ*^2^/*df* = 16.791, CFI = 0.861, TLI = 0.830, RMSEA = 0.154, and SRMR = 0.063. In contrast, the three-factor model showed substantially improved fit, *χ*^2^/*df* = 2.368, CFI = 0.989, TLI = 0.985, RMSEA = 0.045, and SRMR = 0.023. These results indicate that, although common method bias or high item homogeneity should be considered when interpreting the relationships among variables, the one-factor model could not adequately explain the data structure. The three-dimensional structure of ATEM, ET, and ARPI showed better model fit.

### 4.2 Exploratory factor analysis

EFA was conducted using the EFA subsample. The KMO value was 0.948, and Bartlett’s test of sphericity was significant, *χ*^2^(66) = 3039.267, *p* < 0.001, indicating that the data were suitable for factor analysis. The cumulative variance explained by the three rotated factors was 74.149%. Overall, the results supported the three-factor structure. F1, F2, and F3 corresponded to ATEM, ET, and ARPI, respectively.

The item loading results are shown in Table 2. All items loaded most strongly on their corresponding factors, with primary factor loadings ranging from 0.584 to 0.988. No salient cross-loadings were observed. Overall, the EFA results supported the three-dimensional structure of ATEM, ET, and ARPI.

**Table 2:** EFA and CFA item loadings.

Dimension	Item	Brief item description	EFA-F1	EFA-F2	EFA-F3	CFA *λ*
ATEM	ATEM1	Tension before public performance	0.979	−0.124	−0.064	0.753
	ATEM2	Worry about poor performance before performance	0.867	0.101	−0.074	0.819
	ATEM3	Faster heartbeat before performance	0.839	0.019	0.020	0.804
	ATEM4	Physical tension during stage performance	0.742	−0.030	0.213	0.848
	ATEM5	Worry about making mistakes before performance	0.584	0.246	0.107	0.811
ARPI	ARPI1	Difficulty performing normally under anxiety	0.126	−0.038	0.846	0.886
	ARPI2	More likely to make mistakes when nervous	−0.038	−0.068	0.988	0.829
	ARPI3	Anxiety affects musical expression	−0.038	0.172	0.798	0.891
ET	ET1	Concern about others’ evaluation of performance ability	0.080	0.890	−0.081	0.844
	ET2	Poor performance affects others’ views	0.035	0.812	0.074	0.899
	ET3	Feeling loss of face after stage mistakes	0.056	0.816	0.026	0.826
	ET4	Worry that others may think one lacks ability	−0.131	0.946	−0.001	0.831

EFA fixed the extraction at three factors and used promax oblique rotation. F1, F2, and F3 corresponded to ATEM, ET, and ARPI, respectively. CFA *λ* refers to the standardized loading in the three-factor model based on the CFA subsample.

### 4.3 Confirmatory factor analysis

CFA was conducted using the CFA subsample. To examine the internal structure of MPA, this study compared a one-factor model, a two-factor model, and a three-factor model. The results are presented in Table 3. The one-factor model showed poor fit, *χ*^2^/*df* = 8.682, CFI = 0.867, TLI = 0.837, RMSEA = 0.152, and SRMR = 0.061. The fit of the two-factor model improved, but the overall fit was still unsatisfactory, *χ*^2^/df = 6.169, CFI = 0.912, TLI = 0.891, RMSEA = 0.125, and SRMR = 0.053. In contrast, the three-factor model showed good fit, *χ*^2^/df = 1.692, CFI = 0.989, TLI = 0.985, RMSEA = 0.046, and SRMR = 0.025.

**Table 3:** CFA model comparison results.

Sample	Model	N	*χ* ^2^	*df*	*χ*^2^/df	*p*	CFI	TLI	RMSEA	SRMR
CFA subsample	One-factor	332	468.843	54	8.682	<0.001	0.867	0.837	0.152	0.061
	Two-factor	332	326.962	53	6.169	<0.001	0.912	0.891	0.125	0.053
	Three-factor	332	86.277	51	1.692	0.001	0.989	0.985	0.046	0.025
Total sample	One-factor	664	906.720	54	16.791	<0.001	0.861	0.830	0.154	0.063
	Two-factor	664	630.107	53	11.889	<0.001	0.906	0.883	0.128	0.055
	Three-factor	664	120.771	51	2.368	<0.001	0.989	0.985	0.045	0.023

In the one-factor model, all 12 items were specified to load on one general MPA factor. In the two-factor model, the ATEM and ET items were specified to load on one factor, and the ARPI items were specified to load on another factor. In the three-factor model, ATEM, ET, and ARPI were specified as three correlated factors.

To examine the stability of the results, this study further repeated the model comparison using the total sample. The results from the total sample also supported the three-factor model. In the total sample, the one-factor and two-factor models showed unsatisfactory fit, whereas the three-factor model showed good fit, *χ*^2^/df = 2.368, CFI = 0.989, TLI = 0.985, RMSEA = 0.045, and SRMR = 0.023. These results indicate that the three-factor structure consisting of ATEM, ET, and ARPI outperformed the one-factor and two-factor models. Therefore, H1 was supported.

### 4.4 Reliability, convergent validity, and discriminant validity

All three dimensions showed good internal consistency. Cronbach’s *α* values for ATEM, ARPI, and ET were 0.908, 0.897, and 0.906, respectively. The CR values of all three dimensions were above 0.70, and the AVE values were above 0.50, indicating acceptable convergent validity. For discriminant validity, the square root of AVE for each dimension was higher than its maximum correlation with the other dimensions, indicating acceptable discriminant validity among the three dimensions. The results are presented in Table 4.

**Table 4:** Reliability, convergent validity, and discriminant validity.

Dimension	Number of items	*α*	CR	AVE	√AVE	Max. corr.
ATEM	5	0.908	0.904	0.653	0.808	0.737
ARPI	3	0.897	0.903	0.756	0.869	0.737
ET	4	0.906	0.912	0.723	0.850	0.718

Max. corr. = maximum correlation with other dimensions in the CFA subsample.

### 4.5 Correlation analysis and regression analysis

The descriptive statistics, correlation analysis, and regression analysis results for the three dimensions are presented in Table 5. The mean scores of ATEM, ET, and ARPI were 3.432, 3.300, and 3.260, respectively. The correlation analysis showed significant positive correlations among the three dimensions. ATEM was significantly correlated with ET, *r* = 0.706, *p* < 0.001. ATEM was significantly correlated with ARPI, *r* = 0.738, *p* < 0.001. ET was significantly correlated with ARPI, *r* = 0.694, *p* < 0.001. Therefore, H2 was supported.

**Table 5:** Descriptive statistics, correlation analysis, and regression analysis results.

Variable	M	SD	ATEM	ET	ARPI	B	SE	*β*	*t*	VIF
ATEM	3.432	0.893	—			0.454	0.032	0.496	14.347	1.994
ET	3.300	0.803	0.706***	—		0.350	0.035	0.344	9.943	1.994
ARPI	3.260	0.819	0.738***	0.694***	—	—	—	—	—	—

Correlation coefficients are shown in the correlation matrix. The regression analysis used ARPI as the dependent variable and ATEM and ET as independent variables. *R*^2^ = 0.604, adjusted *R*^2^ = 0.603, F(2, 661) = 504.610, *p* < 0.001. ***p < 0.001.

A multiple linear regression analysis was further conducted with ARPI as the dependent variable and ATEM and ET as independent variables. The overall model was significant, *F*(2, 661) = 504.610, *p* < 0.001, *R*^2^ = 0.604, adjusted *R*^2^ = 0.603, indicating that ATEM and ET jointly explained approximately 60.4% of the variance in ARPI. The VIF values for ATEM and ET were both 1.994, indicating no severe multicollinearity. The results showed that ATEM had a significant positive regression coefficient for ARPI, B = 0.454, *β* = 0.496, *t* = 14.347, *p* < 0.001. ET also had a significant positive regression coefficient for ARPI, B = 0.350, *β* = 0.344, *t* = 9.943, *p* < 0.001. These results indicate that higher ATEM and ET were both associated with higher ARPI. Therefore, H3 was statistically supported.

### 4.6 Measurement invariance analysis

Measurement invariance was further examined across the four grouping variables. As shown in Table 6, all configural models demonstrated acceptable fit, with CFI values ranging from 0.9780 to 0.9864, TLI values from 0.9715 to 0.9824, RMSEA values from 0.0494 to 0.0621, and SRMR values from 0.0284 to 0.0376. Changes in CFI, RMSEA, and SRMR across successive models remained within the recommended thresholds. The invariance results therefore supported subsequent group comparisons.

**Table 6:** Measurement invariance results across student groups.

Grouping variable	Model	N	CFI	TLI	RMSEA	SRMR	ΔCFI	ΔRMSEA	ΔSRMR
Gender	Configural	664	0.9864	0.9824	0.0494	0.0284	—	—	—
	Metric	664	0.9860	0.9834	0.0481	0.0319	−0.0004	−0.0014	0.0034
	Scalar	664	0.9855	0.9841	0.0470	0.0334	−0.0005	−0.0010	0.0016
Undergraduate year level	Configural	663	0.9780	0.9715	0.0621	0.0376	—	—	—
	Metric	663	0.9788	0.9758	0.0573	0.0489	0.0008	−0.0049	0.0113
	Scalar	663	0.9782	0.9777	0.0550	0.0482	−0.0006	−0.0023	−0.0007
Major direction	Configural	599	0.9794	0.9734	0.0614	0.0294	—	—	—
	Metric	599	0.9803	0.9766	0.0576	0.0341	0.0009	−0.0038	0.0047
	Scalar	599	0.9799	0.9779	0.0560	0.0356	−0.0004	−0.0016	0.0015
Public performance frequency	Configural	664	0.9802	0.9743	0.0583	0.0323	—	—	—
	Metric	664	0.9790	0.9756	0.0568	0.0426	−0.0012	−0.0015	0.0103
	Scalar	664	0.9767	0.9756	0.0568	0.0445	−0.0023	0.0000	0.0019

Changes in fit indices for the metric model were calculated relative to the configural model, whereas changes for the scalar model were calculated relative to the metric model. Major-direction invariance was examined between vocal music and instrumental music students only.

### 4.7 Group difference analysis

To address RQ1, this study compared ATEM, ET, and ARPI across gender, year level, and public performance frequency, as well as between vocal music and instrumental music students. Group means and standard deviations are presented in Table 7. Descriptively, female students, lower-year undergraduates, instrumental music students, and students with low public performance frequency generally reported higher scores. Because the postgraduate group included only one participant, the year-level analysis included only first- to fourth-year undergraduates.

**Table 7:** Means and standard deviations of ATEM, ET, and ARPI across groups.

Grouping variable	Category	ATEM M ± SD	ET M ± SD	ARPI M ± SD
Gender	Male	3.21 ± 0.88	3.12 ± 0.77	3.14 ± 0.85
	Female	3.54 ± 0.88	3.39 ± 0.81	3.32 ± 0.80
Year level	First year	3.64 ± 0.84	3.43 ± 0.80	3.41 ± 0.82
	Second year	3.56 ± 0.87	3.40 ± 0.80	3.36 ± 0.84
	Third year	3.30 ± 0.81	3.19 ± 0.70	3.15 ± 0.68
	Fourth year	2.97 ± 1.05	3.03 ± 0.97	2.94 ± 0.97
Major direction	Vocal music	3.34 ± 0.90	3.22 ± 0.79	3.17 ± 0.82
	Instrumental music	3.59 ± 0.91	3.44 ± 0.80	3.41 ± 0.80
	Composition/conducting	3.25 ± 0.82	3.28 ± 0.87	3.24 ± 0.88
	Music education or other related areas	3.56 ± 0.68	3.30 ± 0.84	3.32 ± 0.69
Public performance frequency	Low	3.64 ± 0.85	3.41 ± 0.78	3.42 ± 0.81
	Medium	3.05 ± 0.73	3.09 ± 0.77	2.95 ± 0.70
	High	2.76 ± 1.15	2.99 ± 0.94	2.94 ± 1.03

The postgraduate group included only one participant and was therefore not included in the year-level mean comparison or year-level difference analysis. Low = rarely participating in public performance; medium = occasionally participating in public performance; high = frequently participating in public performance.

The difference-test results and effect sizes are presented in Table 8. Female students scored significantly higher than male students on all three dimensions, and instrumental music students scored significantly higher than vocal music students. Significant year-level differences were also found. Games–Howell comparisons showed that first- and second-year students scored significantly higher than third- and fourth-year students on ATEM and ET. For ARPI, first-year students scored significantly higher than third- and fourth-year students, while second-year students scored significantly higher than fourth-year students. Public performance frequency also differed significantly across all three dimensions. *Post hoc* comparisons showed that the low-frequency group scored significantly higher than both the medium- and high-frequency groups on ATEM, ET, and ARPI, whereas the medium- and high-frequency groups did not differ significantly. These findings addressed RQ1.

**Table 8:** Group difference test results.

Grouping variable	Dimension	Test	Statistic	*p*	Effect size	Main difference
Gender	ATEM	*t*-test	−4.615	<0.001	d = 0.382	Female > male
	ET	Welch’s *t*-test	−4.126	<0.001	d = 0.336	
	ARPI	*t*-test	−2.752	0.006	d = 0.228	
Year level	ATEM	Welch’s ANOVA	12.101	<0.001	*η*^2^ = 0.060	Generally higher in Years 1–2
	ET	Welch’s ANOVA	6.592	<0.001	*η*^2^ = 0.032	
	ARPI	Welch’s ANOVA	7.300	<0.001	*η*^2^ = 0.037	
Major direction	ATEM	*t*-test	−3.222	0.001	d = 0.272	Instrumental music > vocal music
	ET	*t*-test	−3.305	0.001	d = 0.279	
	ARPI	*t*-test	−3.562	<0.001	d = 0.301	
Public performance frequency	ATEM	Welch’s ANOVA	43.889	<0.001	*η*^2^ = 0.118	Low > medium/high; medium vs. high, ns
	ET	One-way ANOVA	13.958	<0.001	*η*^2^ = 0.041	
	ARPI	Welch’s ANOVA	28.198	<0.001	*η*^2^ = 0.073	

The postgraduate group was excluded from the year-level difference analysis because it included only one participant. d = Cohen’s d; *η*^2^ = eta squared. Significant omnibus ANOVA results were followed by Games–Howell or Tukey HSD post hoc comparisons, as appropriate. ns = not significant.

## 5 Discussion

This study examined the structural position of ET in MPA among Chinese university music students and its relationships with ATEM and ARPI. The results supported a three-factor structure comprising ATEM, ET, and ARPI. ET was significantly and positively correlated with both ATEM and ARPI. When ATEM and ET were included simultaneously in the regression model, both showed significant positive associations with ARPI. These results indicate that ET is not merely an external correlate of MPA but can also be identified as an important internal dimension of MPA among Chinese university music students.

### 5.1 Evidence for ET as an internal dimension of MPA

One important finding of this study is that ATEM, ET, and ARPI can be distinguished as three correlated dimensions. This result supports H1 and indicates that MPA among Chinese university music students is not a single tension response. Rather, it includes tension and error monitoring before and during performance, image-related concerns in public evaluation, and the interference of anxiety with performance quality and musical expression.

This finding is consistent with previous research on the multidimensional structure of MPA. Kenny (2009) showed in the factor structure study of the K-MPAI that MPA involves components such as somatic anxiety, negative cognition, self- and other-scrutiny, and concerns about performance outcomes. Validation studies among Chinese music students have also suggested that the structure of MPA needs to be further examined in relation to specific cultural and training contexts (Pan et al., 2025; Zhao and Wong, 2026). Building on this work, the present study further shows that evaluative concerns are not only correlates of MPA but can also constitute a distinct dimension within the internal structure of MPA.

The significance of this result lies in its transformation of ET from a general external influencing factor into a testable structural component. Previous studies have often explained MPA from the perspectives of fear of negative evaluation, perfectionism, social anxiety, rumination, self-efficacy, and self-compassion. The present study shows that concerns about others’ evaluations, ability image, and loss of face can constitute part of students’ performance anxiety experience itself. Thus, ET is an important entry point for understanding the structure of MPA among Chinese university music students.

### 5.2 ET in the context of university music training in China

The structural meaning of ET needs to be understood within the context of university music training in China. Professional music training has a continuous evaluative nature. Students need to demonstrate their vocal, instrumental, and musical expression abilities in classroom performance, professional examinations, competitions, and public performances. Evaluations from teachers, peers, judges, and audiences can influence students’ judgments of their ability level, professional position, and future development. Therefore, students’ MPA often contains clear evaluative content.

This finding suggests that evaluative pressure in music performance is not merely a situational background. For university music students, evaluative pressure may form part of their anxiety experiences. Students may worry not only about whether a single performance goes smoothly but also about whether mistakes will affect teachers’ judgments, peer comparisons, performance opportunities, and professional image. ET therefore has educational contextual significance. It reflects students’ concerns about their professional position and ability image during continuous training and public display.

In the Chinese cultural context, ET is also connected with the concept of face. Face concerns social reputation, interpersonal recognition, and one’s position within relationships (Hu, 1944; Hwang, 2011; Zane and Yeh, 2002). Mistakes in music performance are immediate and visible, making them easy for students to interpret as signs of insufficient ability or damaged professional image. This interpretation is illustrated by the lived experiences of tertiary piano students in China. A poor stage performance may lead students to fear that audiences will regard them as insufficiently diligent or technically weak, while public evaluation before expert judges may transform performance difficulties into feelings of shame, inferiority, and diminished self-worth. Unsatisfactory examination or audition results may also be interpreted as letting teachers and parents down, and family judgments concerning talent, educational investment, and professional achievement may intensify this pressure (Miao et al., 2026). These experiences show that, within Chinese university music training, a public mistake reveals more than the quality of technical execution. It also engages teacher approval, evaluation by peers and audiences, and family expectations, thereby connecting performance outcomes with students’ professional image, interpersonal recognition, and relational standing. Therefore, concern over loss of face can serve as a cultural cue of ET. This result suggests that understanding MPA among Chinese university music students requires attention to technical training and students’ interpretations of public evaluation, professional recognition, and loss of face.

### 5.3 Relationship between ET and performance impairment experiences

This study further found that ET was significantly and positively correlated with both ATEM and ARPI, and that both ATEM and ET showed significant positive associations with ARPI in the regression model when included simultaneously. These findings support H2 and H3. They indicate that students’ concerns about public evaluation, ability image, and loss of face are closely related to their perceived decline in performance quality, impaired technical control, and weakened musical expression.

The association between ET and ARPI can be understood from the perspectives of self-presentation and fear of negative evaluation. Students with higher ET may be more likely to interpret performance mistakes as threats to their image and to shift attentional resources toward others’ reactions and error cues (Schlenker and Leary, 1982; Leary, 1983). This attentional bias may be related to students’ perceptions of impaired technical control, performance stability, and musical expression. In other words, evaluative concerns are associated with greater difficulty maintaining stable attention to the musical task itself and with a stronger subjective experience of performance impairment.

Notably, ATEM had a larger standardized regression coefficient for ARPI than ET did (*β* = 0.496 vs. *β* = 0.344). ATEM captures tension, physiological arousal, error monitoring, and self-monitoring immediately before and during performance, whereas ARPI captures disruption to technical control, performance quality, and musical expression within the same performance process. ATEM is therefore more directly connected with the immediate execution difficulties captured by ARPI. By contrast, ET centers on socially evaluative concerns involving others’ judgments, professional image, and loss of face, which are less directly tied to moment-to-moment performance execution. The larger coefficient for ATEM should therefore be interpreted as reflecting its closer functional connection with immediate performance impairment, rather than indicating that ET is unimportant within the structure of MPA.

Beyond these immediate performance processes, the significant association between ET and ARPI also resonates with previous MPA research. Nielsen et al. (2018) found that MPA was related to subjective performance quality and post-event rumination. The present study extends this evidence by showing that perceived decline in performance quality is specifically associated with evaluative concerns. Students with higher ET may be more likely to repeatedly recall others’ reactions and their own mistakes after performance and interpret these experiences as evidence of decreased performance quality. Therefore, the relationship between ET and ARPI has both statistical significance and educational relevance.

### 5.4 Group differences as supplementary evidence for understanding ET

The group difference results provide supplementary evidence for understanding ET. Female students, lower-year undergraduate students, instrumental music students, and students with low public performance frequency generally scored higher on ATEM, ET, and ARPI.

The higher scores among female students are consistent with some studies on gender differences in MPA, but this difference should be interpreted cautiously (Brugués, 2011). The higher scores among lower-year undergraduate students may be related to their recent entry into the university-level professional training system. Facing new teacher standards, peer comparison, and public performance requirements, lower-year undergraduate students may be more likely to experience anticipatory tension, error monitoring, and evaluative concerns.

The higher scores among instrumental music students may be related to the visibility and immediacy of technical errors in instrumental performance. However, because the effect sizes for major direction were small, this result should not be overinterpreted. By contrast, differences by public performance frequency were more pronounced in the present sample. Students with low public performance frequency scored significantly higher than those with medium and high public performance frequencies on all three dimensions, whereas the medium- and high-frequency groups did not differ significantly. In particular, public performance frequency showed the largest observed effect size for ATEM, *η*^2^ = 0.118, indicating relatively stronger practical significance. This suggests that insufficient public performance experience may be associated with stronger anticipatory tension, error monitoring, and evaluative concerns.

Music performance training should address evaluative pressure, error coping, and the accumulation of stage experience alongside technical improvement. Music training should gradually increase opportunities for public performance to help students manage evaluative pressure and performance difficulties. Teachers should also provide supportive feedback and reduce excessive peer comparison.

## 6 Implications and limitations

### 6.1 Theoretical implications

This study has three main theoretical contributions. First, it supported a three-dimensional structure comprising ATEM, ET, and ARPI, providing preliminary psychometric evidence for assessing MPA among Chinese university music students. Second, it supported the status of ET as an internal dimension, indicating that evaluative concerns are part of the structure of MPA. Third, it incorporated concern over loss of face into the explanation of ET, making the study of MPA among Chinese university music students more closely connected to specific cultural contexts.

These contributions are grounded in the empirical results of this study. The three-factor structure indicates that MPA can be divided into anticipatory tension and error monitoring, evaluative threat, and anxiety-related performance impairment. The correlation and regression results show that ET is closely related to both ATEM and ARPI. The group difference results further show that the three dimensions of MPA vary across different student groups.

### 6.2 Practical implications

The findings suggest that performance-related psychological support in university music training should pay greater attention to ET. ET was significantly related to ARPI, indicating that students’ concerns about others’ evaluations, ability image, and loss of face are closely connected with their performance impairment experiences. Therefore, in public performance settings, teachers may need to pay attention to how students interpret evaluative information. Feedback that is specific, constructive, and improvement-oriented may help reduce students’ concerns about damage to their professional image.

The group difference results suggest that lower-year students and students with low public performance frequency may need more performance-related psychological support. Lower-year students scored higher overall on the three dimensions, indicating that performance-related psychological support can be introduced earlier in professional training. Students with low public performance frequency scored significantly higher than those with medium and high public performance frequencies on ATEM, ET, and ARPI, and the effect size of public performance frequency was particularly notable for ATEM. This suggests that performance exposure has practical significance. Universities may consider providing students with gradual stage experiences that move from low-pressure to higher-pressure settings. Studies on coping strategies and exposure training also suggest that repeated contact with performance situations can help students accumulate coping experience (Bakhtiari et al., 2025; Zhang et al., 2026).

Instrumental music students scored higher overall than vocal music students on ATEM, ET, and ARPI, suggesting that this group deserves further attention. Instrumental performance usually requires high levels of technical accuracy, rhythmic control, and on-stage stability. Students may also be more likely to interpret wrong notes, unstable rhythm, or interruptions as visible mistakes. Teaching practice may therefore include error-recovery exercises, continuation exercises after mistakes, and on-stage attentional regulation training to help students develop more stable stage coping experience. Overall, this study suggests that music departments can incorporate teacher feedback methods, stage adaptation training, and performance-related psychological support into regular professional training, allowing psychological support to enter students’ learning process earlier and more continuously (Araújo et al., 2017; Hildebrandt et al., 2012).

### 6.3 Limitations

This study has four limitations. First, it used a cross-sectional questionnaire design in which all variables were measured at a single time point. Therefore, the temporal order among ATEM, ET, and ARPI cannot be established. ATEM and ET were significantly related to ARPI in the regression model, but these results indicate statistical associations rather than causality. They do not demonstrate that either ATEM or ET directly causes performance impairment. Future studies may use longitudinal designs to examine whether the three dimensions change with training stage and performance experience.

Second, all data were collected through self-report questionnaires, which may involve common method bias. Harman’s single-factor test showed that the first unrotated factor explained 62.785% of the total variance, indicating that this risk should be taken seriously. The CFA results showed that the three-factor model clearly outperformed the one-factor model. Even so, the use of the same self-report method for all variables may have inflated the observed correlations and regression coefficients. Future research may further validate the findings by incorporating teacher evaluations, behavioral observations, physiological indicators, or longitudinal data.

Third, the sample structure was not fully balanced. Female students accounted for a relatively high proportion of the sample. The postgraduate group was very small, the number of students with high public performance frequency was limited, and the sample sizes of composition/conducting and music education or other related areas were also small. These factors may affect the stability of the group difference results. Future studies should expand the sample sources and improve the balance among groups with different year levels, major directions, and performance frequencies.

Fourth, this study used a brief 12-item context-specific measure. Although the EFA, CFA, reliability, and validity results supported its basic structure, the instrument still needs to be further validated across more regions, different types of universities, and different music student groups. Future research may also compare this three-dimensional structure with mature instruments such as the K-MPAI and K-MPAI-R to further examine its external validity.

## 7 Conclusion

Based on questionnaire data from 664 Chinese university music students, this study examined the structural position of ET in MPA. The results supported the interpretation of ATEM, ET, and ARPI as three related dimensions of MPA. Model comparisons in both the CFA subsample and the total sample showed that the three-factor model outperformed the one-factor and two-factor models. Measurement invariance analyses further supported configural, metric, and scalar invariance across gender, year level, and public performance frequency, as well as between vocal music and instrumental music students. This indicates that MPA among Chinese university music students cannot be reduced to general performance tension. Rather, it should be understood as a multidimensional structure involving anticipatory tension and error monitoring, evaluative threat, and anxiety-related performance impairment.

The core finding of this study is that ET can serve as an internal dimension of MPA. Students’ concerns about others’ evaluations, their ability image, and loss of face constitute important content of their performance anxiety experience. The correlation and regression results further showed that ET had significant positive associations with both ATEM and ARPI. This indicates that ET is closely related to students’ perceived performance impairment experiences.

The group difference results showed that female students, lower-year students, instrumental music students, and students with low public performance frequency generally scored higher on all three dimensions. Among these group comparisons, differences by public performance frequency were more pronounced in the present sample. Students with low public performance frequency scored significantly higher on ATEM, ET, and ARPI than those with medium and high public performance frequencies, whereas the medium- and high-frequency groups did not differ significantly.

By incorporating ET into the internal structure of MPA among Chinese university music students, this study supplements previous research perspectives that have treated evaluative pressure mainly as an external influencing factor. The findings provide an explanation of performance anxiety among Chinese university music students that is more closely connected to training contexts and cultural contexts. They also provide empirical evidence for future performance-related psychological support, improvement of evaluative feedback, and gradual stage exposure training.

## Data Availability

The original contributions presented in the study are included in the article/Supplementary material, further inquiries can be directed to the corresponding author.

## Footnotes

1
https://spssau.com/indexs.html
